# Characteristics of *N*^6^-methyladenosine Modification During Sexual Reproduction of *Chlamydomonas reinhardtii*

**DOI:** 10.1016/j.gpb.2022.04.004

**Published:** 2022-05-10

**Authors:** Ying Lv, Fei Han, Mengxia Liu, Ting Zhang, Guanshen Cui, Jiaojiao Wang, Ying Yang, Yun-Gui Yang, Wenqiang Yang

**Affiliations:** 1 Photosynthesis Research Center, Key Laboratory of Photobiology, Institute of Botany, Chinese Academy of Sciences, Beijing 100093, China; 2 China National Botanical Garden, Beijing 100093, China; 3 College of Advanced Agricultural Sciences, University of Chinese Academy of Sciences, Beijing 100049, China; 4 CAS Key Laboratory of Genomic and Precision Medicine, Beijing Institute of Genomics, Chinese Academy of Sciences and China National Center for Bioinformation, Beijing 100101, China; 5 Sino-Danish College, University of Chinese Academy of Sciences, Beijing 100049, China; 6 Institute of Stem Cell and Regeneration, Chinese Academy of Sciences, Beijing 100101, China; 7 Innovative Academy of Seed Design, Chinese Academy of Sciences, Beijing 100093, China

**Keywords:** *N*^6^-methyladenosine, m^6^A sequencing, Sexual reproduction, Microtubule-associated pathway, Photosynthesis, *Chlamydomonas reinhardtii*

## Abstract

The unicellular green alga ***Chlamydomonas reinhardtii*** (hereafter *Chlamydomonas*) possesses both plant and animal attributes, and it is an ideal model organism for studying fundamental processes such as **photosynthesis**, **sexual reproduction**, and life cycle. ***N***^**6**^**-methyladenosine** (m^6^A) is the most prevalent mRNA modification, and it plays important roles during sexual reproduction in animals and plants. However, the pattern and function of m^6^A modification during the sexual reproduction of *Chlamydomonas* remain unknown. Here, we performed transcriptome and methylated RNA immunoprecipitation sequencing (MeRIP-seq) analyses on six samples from different stages during sexual reproduction of the *Chlamydomonas* life cycle*.* The results show that m^6^A modification frequently occurs at the main motif of DRAC (D = G/A/U, R = A/G) in *Chlamydomonas* mRNAs. Moreover, m^6^A peaks in *Chlamydomonas* mRNAs are mainly enriched in the 3′ untranslated regions (3′UTRs) and negatively correlated with the abundance of transcripts at each stage. In particular, there is a significant negative correlation between the expression levels and the m^6^A levels of genes involved in the **microtubule-associated pathway**, indicating that m^6^A modification influences the sexual reproduction and the life cycle of *Chlamydomonas* by regulating microtubule-based movement. In summary, our findings are the first to demonstrate the distribution and the functions of m^6^A modification in *Chlamydomonas* mRNAs and provide new evolutionary insights into m^6^A modification in the process of sexual reproduction in other plant organisms.

## Introduction

In eukaryotes, sexual reproduction consists of two sequential events: haploid gametes fusing to form diploid zygotes after fertilization and diploid zygotes producing haploid progenies by meiosis [1]. Unlike animals and plants, the unicellular green alga *Chlamydomonas reinhardtii* (hereafter *Chlamydomonas*) has unicellular haploid and diploid bodies like the gametophytes and sporophytes of land plants, respectively [1], [2], [3]. *Chlamydomonas* is known as “Green Yeast”, and it has been introduced as a model organism to study fundamental processes such as photosynthesis, nutrient metabolism, flagella biology, cell cycle, and sexual reproduction [4], [5], [6], [7], [8], [9], [10]. Because *Chlamydomonas* possesses both animal and plant features, studying the cell cycle and sexual reproduction of *Chlamydomonas* can yield important insights into evolution [1]. Many studies have shown that its mitotic cell cycle has a long G1 phase and rapidly alternating S/M phases, which allow *Chlamydomonas* to produce 2^*n*^ (*n* = 1–5) daughter cells in one cell cycle [7], [11]. In *Chlamydomonas*, the cell cycle is mainly controlled by two major cyclin-dependent kinases (CDKs), namely CDKA1 and CDKB1 [12], [13], and other critical proteins reported previously [7]. The *Chlamydomonas* vegetative cells can be induced by nitrogen deficiency to produce two kinds of isogametes: mating type minus (mt−) and mating type plus (mt+) [14]; further, many mating type-specific genes are induced during this process [15], [16], [17]. Gamete-specific agglutinins encoded by the mt+-specific gene (*SAG1*) and the mt−-specific gene (*SAD1*) facilitate the interactions between two different mating type gametes [18]. Different mating type gametes adhere together to initiate the zygote formation process, which includes increased cAMP levels as a signal, flagellar tip activation, loss of the cell wall, and mating structure activation accompanied by actin polymerization [19], [20], [21], [22]. After clumping together, the mating type-specific structures are formed, and this process is controlled by gamete fusion protein 1 (FUS1) in mt+ gametes and hapless 2 (HAP2)/generative cell specific 1 (GCS1) in mt− gametes, respectively [21], [23]. Simultaneously, the expression of zygote-specific genes such as *early zygote expressed* (*EZY*) genes is up-regulated [24], and the transcription factors gamete-specific homeodomain protein (GSP1) in mt+ gametes and gamete-specific minus 1 (GSM1) in mt− gametes accumulate to regulate the transition from haploid cells to the diploid zygotes [2], [3], [8], [17].

In eukaryotes, RNA modifications are crucial for the fate determination of RNA [25]. As the most prevalent regulator found within eukaryotic mRNAs, m^6^A modification is involved in mRNA alternative splicing, nuclear export, stability, translation, and degradation [26], [27], [28], [29], [30], [31]. In mammals, m^6^A modification is catalyzed by a large RNA methyltransferase complex (MTase) as writer proteins, which is composed of methyltransferase-like 3 (METTL3), methyltransferase-like 14 (METTL14), Wilms’ tumor 1-associating protein (WTAP), virilizer like m^6^A methyltransferase associated protein (VIRMA), Casitas B-lineage lymphoma-transforming sequence-like protein 1 (CBLL1, also known as HAKAI), RNA-binding protein 15/15B (RBM15/15B), and zinc finger CCCH domain-containing protein 13 (ZC3H13) [32], [33], [34], [35], [36], [37], [38], [39]. The removal of methyl groups is performed by two RNA demethylases that act as eraser proteins: alkylated DNA repair protein alkB homolog 5 (ALKBH5) and fat mass and obesity-associated protein (FTO) [40], [41], [42]. Furthermore, the functions of m^6^A modification in mammalian mRNA metabolic processes mainly depend on diverse reader proteins, including YTH domain-containing family (YTHDF1–3 and YTHDC1–2), heterogeneous nuclear ribonucleoproteins (HNRNPC, HNRNPG, and HNRNPA2B1), and IGF2 mRNA binding protein family (IGF2BP1–3) [26], [29], [30], [43], [44], [45], [46]. Malfunctions of these proteins cause disorders in m^6^A modification and further affect spermatogenesis and embryo development in animals [42], [47], [48], [49]. Consistently, m^6^A modification is also detected in plants, and their counterparts of animal writers, erasers, and readers have also been identified to play critical roles [50], [51], [52]. Functional studies have shown that m^6^A is also important for embryo development and sporogenesis in plants [50], [53], [54], [55], [56]. These findings suggest that m^6^A modification may have critical conserved roles in the sexual reproduction of mammals and plants [42], [49], [53]. However, whether and how m^6^A modification is involved in sexual reproduction and life cycle regulation of *Chlamydomonas* remains unknown.

Here, we first performed methylated RNA immunoprecipitation sequencing (MeRIP-seq) to depict the m^6^A modification landscapes on six samples (mt+ vegetative cells, mt− vegetative cells, mt+ gametes, mt− gametes, zygotes at day 1, and zygotes at day 7) during the sexual life cycle of *Chlamydomonas* with two biological replicates, and RNA sequencing (RNA-seq) was conducted simultaneously to analyze the associated transcriptional variation. The results show that m^6^A peaks appear widely in *Chlamydomonas* mRNAs, while m^6^A peaks are mainly enriched in the 3′ untranslated regions (3′UTRs) of mRNAs. DRAC (where D represents G/A/U, R represents A/G, and A represents m^6^A) is the main motif of the m^6^A modification peak that occurs among different stages. Moreover, the combined analyses of MeRIP-seq and RNA-seq show that *Chlamydomonas* m^6^A modification is negatively correlated with the abundance of transcripts. In particular, the genes involved in the microtubule-associated pathway display significantly negative correlations between gene expression and m^6^A modification level, suggesting that m^6^A modification regulates microtubule-based movement during sexual reproduction in the *Chlamydomonas* life cycle. Finally, CrMETTL3 and CrMETTL14 are potential m^6^A methyltransferases responsible for m^6^A formation in *Chlamydomonas*. Overall, our findings reveal the distribution of m^6^A modification and its potential regulatory functions during sexual reproduction and the life cycle of *Chlamydomonas*.

## Results

### Features of critical periods during the *Chlamydomonas* life cycle

**Figure 1:**
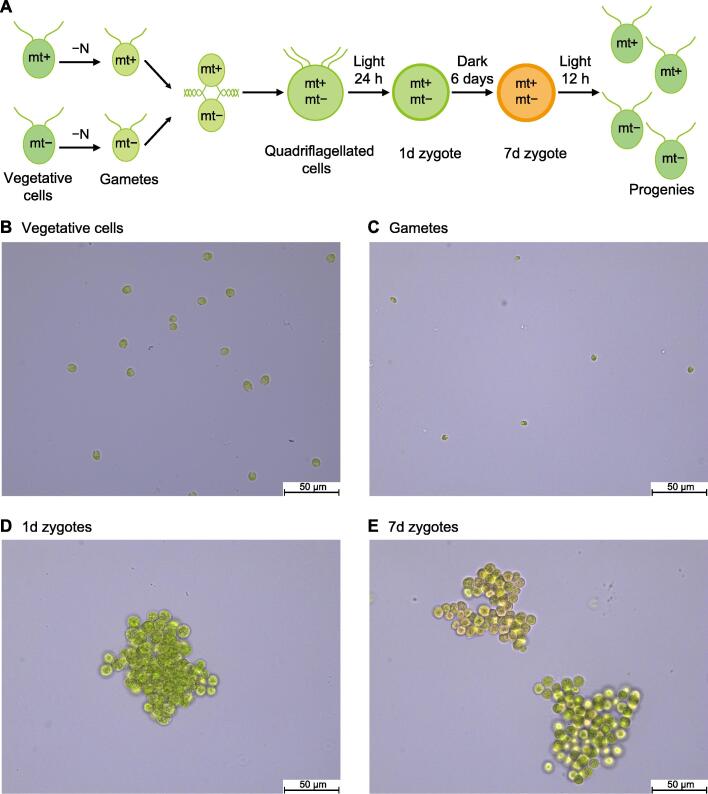
**Sexual life cycle of *Chlamydomonas* and the features of its critical periods** **A.** Overview of the life cycle of *Chlamydomonas.* Vegetative cells can differentiate into gametes in response to nitrogen deficiency; zygotes can be formed from the mating of gametes with different mating types under light and eventually mature over a few days without light; mature zygotes conversely can germinate to form four daughter cell progenies from the tetrad by the addition of nitrogen and light. Image showing the life cycle at different stages. Vegetative cells (**B**), gametes (**C**), 1d zygotes (**D**), and 7d zygotes (**E**) are shown under a 400× light microscope. “−N” indicates nitrogen deficientcy.

To examine the dynamics of m^6^A modification during sexual reproduction of the *Chlamydomonas* life cycle, six samples from key periods, including mt+ vegetative cells, mt− vegetative cells, mt+ gametes, mt− gametes, zygotes at day 1  (1d zygotes), and zygotes at day 7  (7d zygotes), were collected for MeRIP-seq analysis (Figure 1A). During asexual reproduction, cells mainly undertake vegetative growth and mitosis, and the vegetative cells at this stage have classical morphological characteristics: diameters of around 5–7 μm, with two flagella, one cup-shaped chloroplast, an eyespot, a nucleus, and other organelles (Figure 1B). The vegetative cells can be induced to form gametes by nitrogen starvation or blue light, which is called gametogenesis [57]. The gametogenesis of *Chlamydomonas* is presumed to be a stress response, as the gametes show high motility and low photosynthetic activity. It should be noted that gametes are much smaller than vegetative cells (Figure 1C). When induced gametes of the opposite mating types are mixed together to form zygotes, the mating responses are triggered rapidly, followed by the adhesion of mating type-specific agglutinins on the surface of flagella [58]. Compared with the vegetative cells, zygotes without flagella are larger with thicker cell walls (Figure 1D and E). After 1 day in the light and 6 days in the dark, the zygote develops into a zygospore, which is more resistant to various stresses. In favorable environments, the zygospores germinate, and meiosis produces haploid vegetative cells.

### **m^6^A** modification changes dynamically during the sexual reproduction of ***Chlamydomonas***

**Figure 2:**
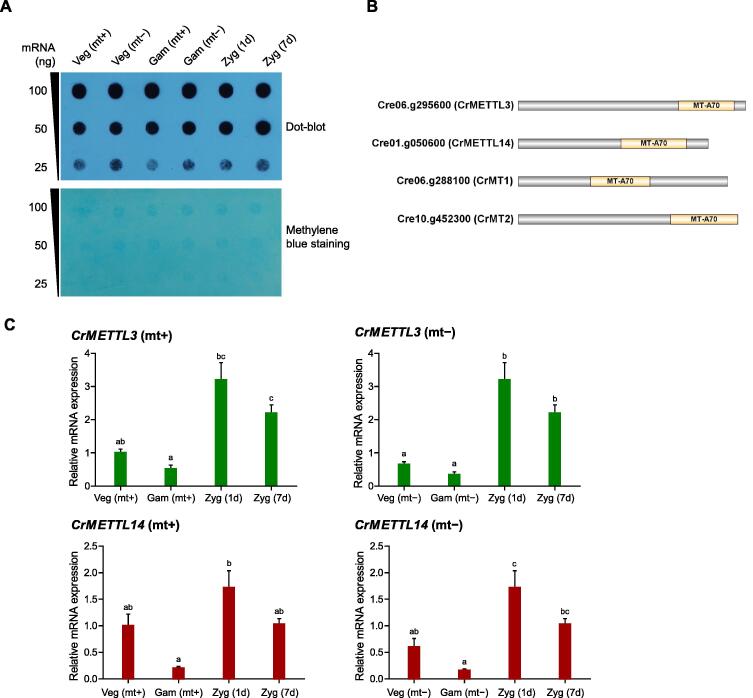
**mRNA m^6^A methylation shows dynamic changes during the life cycle of *Chlamydomonas*** **A.** The overall levels of m^6^A mRNA modification were detected by dot-blot assays using a specific anti-m^6^A antibody (upper panel) and methylene blue staining to show the loading control (lower panel). **B.** Schematic representations of the putative candidates of m^6^A methyltransferases in *Chlamydomonas*. MT-A70 represents the conserved motif. **C.** qRT-PCR shows the relative abundance of transcripts of the putative genes encoding m^6^A methyltransferases in *Chlamydomonas*. The *CBLP* gene was used as the internal control. Error bars represent the standard deviation of three biological replicates. Different lowercase letters over the bars show a significant difference via one-way ANOVA followed by Tukey’s post hoc test by SPSS statistics software (*P* < 0.05). Veg (mt+), mating type plus vegetative cell; Veg (mt−), mating type minus vegetative cell; Gam (mt+), mating type plus gamete; Gam (mt−), mating type minus gamete; Zyg (1d), 1d zygote; Zyg (7d), 7d zygote; qRT-PCR, quantitative real-time PCR.

RNA m^6^A modification is critical for the sexual reproduction of mammals and plants [42], [47], [48], [49], [53], [59]. To examine the levels of m^6^A modification during sexual reproduction in the *Chlamydomonas* life cycle, a dot-blot assay was performed at different stages as described above. The results showed that m^6^A modification was down-regulated during gametogenesis and up-regulated in zygote development, especially in mt+ samples (Figure 2A). Based on the presence of predicted RNA adenosine methylase domains (MT-A70) and full-length human METTL3 and METTL14 protein sequences, four candidate *Chlamydomonas* m^6^A methyltransferases were found in the *Chlamydomonas* phytozome (https://phytozome-next.jgi.doe.gov/), namely CrMETTL3 (Cre06.g295600), CrMETTL14 (Cre01.g050600), CrMT1 (Cre06.g288100), and CrMT2 (Cre10.g452300) (Figure 2B). Among them, CrMT1 and CrMT2 showed lower homology to human METTL3 and METTL14 overall. Quantitative real-time PCR (qRT-PCR) showed that *CrMETTL3* and *CrMETTL14* expression levels consistently declined in gametes with different mating types, increased in 1d zygotes, and then declined again in 7d zygotes (Figure 2C), suggesting that m^6^A modification may participate in regulating sexual reproduction and changes dynamically in *Chlamydomonas*.

### Overview of the **m^6^**A methylome in ***Chlamydomonas***

**Figure 3:**
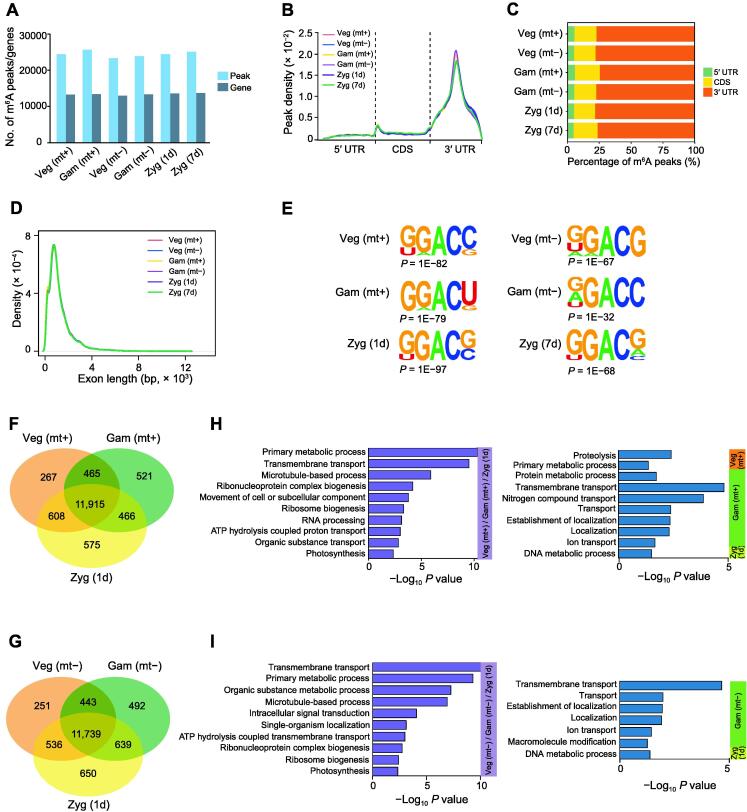
**Overview of the m^6^A methylome in *Chlamydomonas*** **A.** Histogram showing the numbers of the detected m^6^A peaks and methylated genes at each stage of the *Chlamydomonas* life cycle. **B.** Metagene profiles showing the distribution of m^6^A peaks along the transcripts composed of three rescaled non-overlapping segments (5′ UTR, CDS, and 3′ UTR). **C.** Stacked bar chart showing the percentage of m^6^A peaks within distinct RNA sequence types. **D.** Density plot showing the distribution of m^6^A peaks across the length of the exon. **E.** Top sequence motifs identified within m^6^A peaks. **F.** and **G.** Venn diagrams showing the overlap of m^6^A-modified genes among the mt+ vegetative cells, mt+ gametes, and 1d zygotes (F) and among the mt− vegetative cells, mt− gametes, and 1d zygotes (H), respectively. **H.** and **I.** Bar plots showing the GO enrichment of commonly m^6^A-modified genes (left) and specifically m^6^A-modified genes (right) among the mt+ vegetative cells, mt+ gametes, and 1d zygotes (H) and among the mt− vegetative cells, mt− gametes, and 1d zygotes (I), respectively. UTR, untranslated region; CDS, coding sequence; GO, Gene Ontology.

**Supplementary figure S1:**
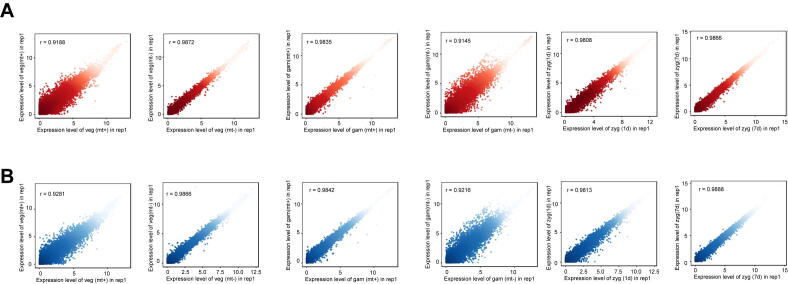
**Pearson correlation among the independent biological replicates**. Pearson correlation analysis shows the high gene expression correlation among independent biological replicates in RNA-seq (A) and MeRIP-seq (B). MeRIP, methylated RNA immunoprecipitation sequencing.

To study the potential role of m^6^A modification in regulating *Chlamydomonas* sexual reproduction, MeRIP-seq was performed on the samples from different stages to compare their transcriptome-wide m^6^A methylomes. Pearson correlation analysis suggested good reproducibility among each group (Figure S1A and B). Further analysis revealed 24,416, 25,656, 23,288, 23,836, 24,403, and 25,057 m^6^A peaks within 13,255, 13,367, 12,969, 13,313, 13,564, and 13,720 transcripts from mt+ vegetative cells, mt+ gametes, mt− vegetative cells, mt− gametes, 1d zygotes, and 7d zygotes, respectively (Figure 3A; Table S1). The distribution pattern of m^6^A modification along the transcripts was analyzed, and the results of metagene profiles revealed that m^6^A deposition was primarily enriched in the 3′ UTR (Figure 3B), which is interestingly consistent with the m^6^A distribution patterns in rice, potato, and maize [55], [60], [61]. We then analyzed the distribution of m^6^A peaks within three non-overlapping regions: 5′ UTR, coding sequence (CDS), and 3′ UTR. Among them, m^6^A peaks appeared to be greatly enriched in the 3′ UTR segment (Figure 3C), with 73%–76% of the peaks falling into this region. Furthermore, the distribution density plot of m^6^A peaks across the exon length showed that m^6^A peaks tend to occur within exons around 760 bp in length (Figure 3D), indicating that the m^6^A modification tends to be catalyzed on long exons while the average length of exons in *Chlamydomonas* is 376.62 bp.

To identify the consensus sequence and enrichment of m^6^A peaks appearing in the transcriptome, HOMER was used to conduct a motif search of high-confidence m^6^A peaks. The DRAC motif (Figure 3E), which is a conserved m^6^A motif found in *Arabidopsis* [50], [62] and other eukaryotes [63], was identified in all of the detected stages, including vegetative cells, gametes, and 1d and 7d zygotes. We next compared the transcriptome-wide m^6^A methylome during sexual reproduction. We found that 11,915 m^6^A-modified genes were shared among mt+ vegetative cells, mt+ gametes, and 1d zygotes, and 11,739 m^6^A-modified genes were shared among mt− vegetative cells, mt− gametes, and 1d zygotes (Figure 3F and G). Less than 10% of the specific m^6^A-modified genes were detected in all stages. To further analyze the regulatory mechanisms of m^6^A modification during sexual reproduction, we then performed a Gene Ontology (GO) analysis of the genes with m^6^A modifications. The commonly m^6^A-modified genes in mt+ vegetative cells, mt+ gametes, and 1d zygotes were enriched in the primary metabolic process, transmembrane transport, and RNA processing (Figure 3H), implying that m^6^A is essential for basic life activities in *Chlamydomonas*. m^6^A modification was also found to be related to the microtubule-based process and photosynthesis, which influence gametogenesis and zygote development during sexual reproduction. Interestingly, the processes enriched by common m^6^A-modified genes in mt− vegetative cells, mt− gametes, and 1d zygotes were similar to the previous findings in mt+ cells (Figure 3I). Genes with specific m^6^A methylation at various stages were related to proteolysis and DNA metabolic process (Figure 3H and I), indicating that m^6^A methylation generally appears in various stages and is involved in the regulation of important processes during the sexual reproduction of *Chlamydomonas*.

### **m^6^A** modification is generally negatively correlated with gene expression level

**Figure 4:**
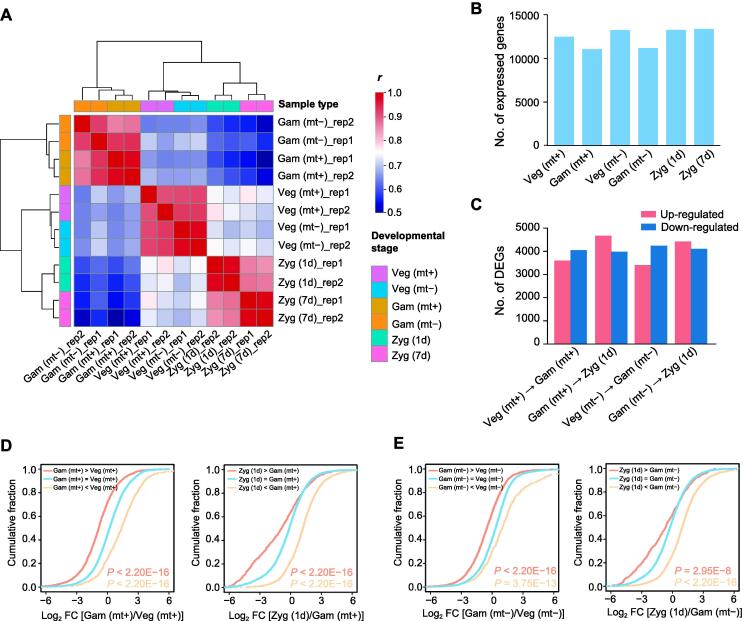
**m^6^A modification is generally negatively correlated with gene expression level** **A.** Heatmap showing the high correlation among replicates based on the expression matrix. **B.** Histogram showing the number of genes expressed at each stage of the *Chlamydomonas* life cycle. **C.** Histogram showing the number of differentially expressed (up-regulated and down-regulated) genes at different stages. **D.** Cumulative distribution displaying the abundance changes in mRNA classified by m^6^A methylation level during transitions from mt+ vegetative cells to mt+ gametes (left) and from mt+ gametes to 1d zygotes (right). In the left panel, Gam (mt+) > Veg (mt+) means up-m^6^A genes, Gam (mt+) = Veg (mt+) means stable-m^6^A genes, and Gam (mt+) < Veg (mt+) means down-m^6^A genes. In the right panel, Zyg (1d) > Gam (mt+), Zyg (1d) = Gam (mt+), and Zyg (1d) < Gam (mt+) mean up-m^6^A, stable-m^6^A, and down-m^6^A genes, respectively. **E.** Cumulative distribution displaying the abundance changes in mRNA classified by m^6^A methylation level during transitions from mt− vegetative cells to mt− gametes (left) and from mt− gametes to 1d zygotes (right). In the left panel, Gam (mt−) > Veg (mt−) means up-m^6^A genes, Gam (mt−) = Veg (mt−) means stable-m6A genes, and Gam (mt−) < Veg (mt−) means down-m^6^A genes. In the right panel, Zyg (1d) > Gam (mt−), Zyg (1d) = Gam (mt−), and Zyg (1d) < Gam (mt−) mean up-m^6^A, stable-m^6^A, and down-m^6^A genes, respectively. *P* values were calculated using two-sided Wilcoxon and Mann-Whitney tests. DEG, differentially expressed gene; FC, fold change.

**Supplementary figure S2:**
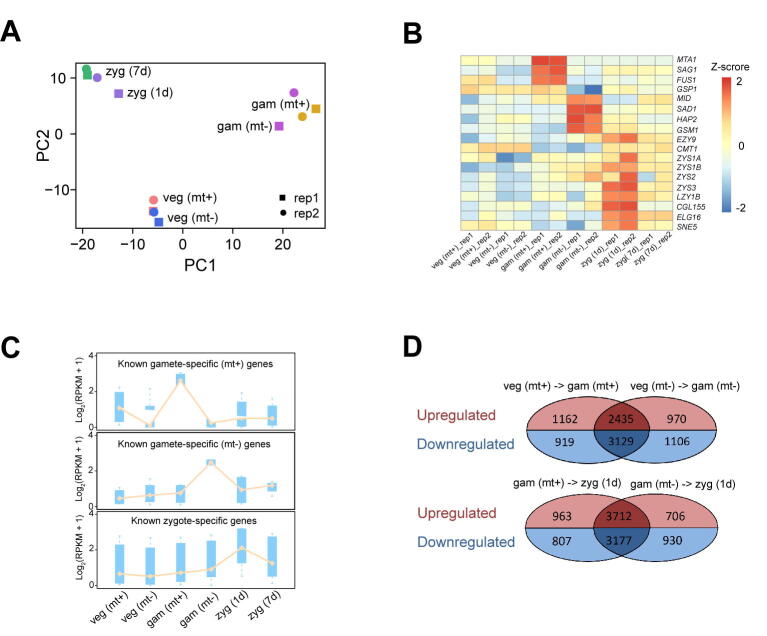
**The profiles of gene expression during the *Chlamydomonas* life cycle**. **A**. PCA based on the gene expression exhibiting the high consistency among replicates. **B**. Heatmap showing the stage-specific expression of some of the differentially expressed genes. **C**. Line chart and box plot of gene expression profiles for reported gamete and zygote specific gene. **D**. Overlap of the upregulated and downregulated expressed genes between mating type minus and plus in the processes of vegetative cells transitioning to gametes (top) and gametes to zygotes (bottom). PCA, principal component analysis; RPKM, reads per kilobase per million mapped.

m^6^A has been proven to regulate the stability of mRNA [29], [64], [65]. According to the findings of the m^6^A methylome at different stages, we further examined the gene expression level to investigate the role of m^6^A regulation in mRNA abundance during sexual reproduction. We determined mRNA abundance as previously described and obtained a transcriptome-wide RNA expression map with a strong correlation between biological replicates (Figure 4A, Figure S2A). Briefly, 12,465, 11,043, 13,229, 11,166, 13,241, and 13,347 stably expressed transcripts were obtained from mt+ vegetative cells, mt+ gametes, mt− vegetative cells, mt− gametes, 1d zygotes, and 7d zygotes, respectively (Figure 4B; Table S2). The expression of some stage-specific genes was also determined (Figure S2B and C), including the well-known *GSP1* (Cre02.g109650) and *GSM1* (Cre08.g375400), which encode transcription factors specifically expressed in mt+ and mt− gametes, respectively, and are involved in the development of zygotes [15], [16], [17]. Moreover, early zygote-specific genes were also identified, including *early zygote expressed 9* (*EZY9*, Cre06.g304500), *zygote-specific 2* (*ZYS2/MAW1*, Cre07.g325812), and *early zygote expressed 3* (*EZY3*, Cre11.g482650), in 1d zygotes [24].

We next analyzed the differentially expressed genes (DEGs) to investigate the transcriptional changes during sexual reproduction. A total of 7645 and 7640 transcripts were differentially expressed (|log_2_ fold change| > 1; FDR < 0.01) between vegetative cells and gametes with mt+ and mt−, respectively (Figure 4C; Table S3), among which 4048 and 4235 genes were down-regulated in mt+ and mt− gametes, respectively. Additionally, we identified 8525 DEGs between mt+ gametes and 1d zygotes, and 8701 genes between mt− gametes and 1d zygotes. Among them, 4675 and 4418 genes exhibited higher expression levels in 1d zygotes than in mt+ and mt− gametes, respectively. We next compared the DEGs between mt+ and mt−. The results (Figure S2D) showed that more than 60% of DEGs were shared between the mating types during transitions from vegetative cells to gametes and from gametes to zygotes.

To study the relationship between the changes in mRNA abundance and m^6^A methylation, the m^6^A-modified genes were classified into three subgroups: genes with increased methylation levels, decreased methylation levels, and stable methylation levels during the reproduction. In the process of mt+ vegetative cells transitioning to mt+ gametes, genes were divided into three types, including up-m^6^A, down-m^6^A, and stable-m^6^A subgroups, respectively, based on the m^6^A changes in mt+ gametes. The mRNA abundance changes in these three subgroups showed greatly contrasting variability. Compared with stable-m^6^A genes, the mRNA abundance of up-m^6^A genes in mt+ gametes tend to be down-regulated, while down-m^6^A genes generally exhibit significantly higher mRNA abundance (Figure 4D, left). These results suggested that mRNA abundance is negatively regulated by m^6^A methylation in the mt+ gametogenesis. The negative correlation between m^6^A and mRNA abundance was also observed in other samples from different stages, including mt− gametogenesis (Figure 4E, left), mt+ gametes transitioning to 1d zygotes (Figure 4D, right), and mt− gametes transitioning to 1d zygotes (Figure 4E, right), suggesting that m^6^A modification negatively regulate mRNA abundance during the sexual reproduction of *Chlamydomonas*.

### **m^6^A** is involved in the sexual reproduction by regulating microtubule-based movement

**Figure 5:**
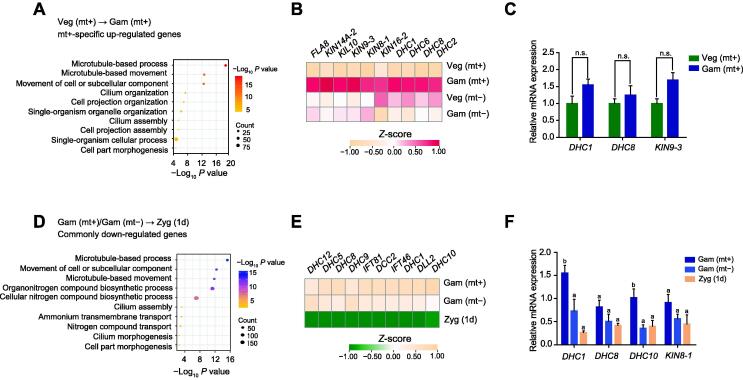
**m^6^A is involved in the life cycle by regulating microtubule-based movement** **A.** GO enrichment of the up-regulated genes in mt+ gametes during gametogenesis. **B.** Heatmap of up-regulated genes associated with the microtubule-based process during gametogenesis. **C.** qRT-PCR analysis showing the expression levels of the target genes regulated by m^6^A during gametogenesis. The *CBLP* gene was used as the internal control. Error bars represent the standard deviation of three biological replicates. n.s., not significant. **D.** GO enrichment of the down-regulated genes in 1d zygotes during the gamete fusion process. **E.** Heatmap of down-regulated genes associated with the microtubule-based process during the gamete fusion process. **F.** qRT-PCR to validate the target genes regulated by m^6^A during gamete fusion. The *CBLP* gene was used as the internal control. Error bars represent the standard deviation of three biological replicates. Different lowercase letters over the bars show a significant difference via one-way ANOVA followed by Tukey’s post hoc test by SPSS statistics software (*P* < 0.05).

**Supplementary figure S3:**
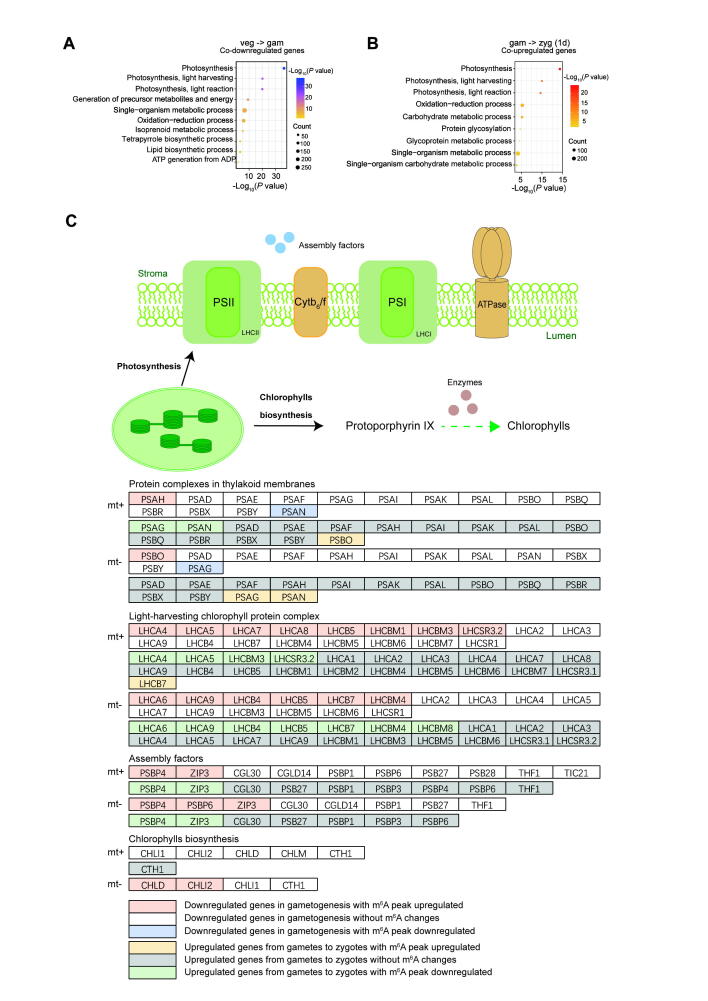
**Biological pathways related to the Chlamydomonas life cycle. A**. GO enrichment of downregulated genes in both gametes with different mating types (mt+ and mt-) during gametogenesis. **B**. GO enrichment of upregulated genes in 1d zygotes in the transitions of mt+ and mt- gametes to zygotes. **C**. The m6A modification level of photosynthesis is associated with genes during sexual reproduction. GO, gene ontology.

To further explore the role of m^6^A in sexual reproduction, we investigated biological processes related to sexual reproduction and analyzed whether m^6^A could negatively regulate the expression levels of key genes involved. GO analyses showed that most down-regulated genes during mt+ and mt− gametogenesis were enriched in photosynthesis-associated processes (Figure S3A and C). We speculate that vegetative cells need to reduce photosynthesis in response to nitrogen starvation and undergo gametogenesis. Most up-regulated genes from mt+ vegetative cells transitioning to mt+ gametes were specifically enriched in the microtubule-based process and cilium organization (Figure 5A). Previous research has reported that genes associated with microtubule-based processes encode kinesin and dynein proteins, which mediate the intraflagellar transport system, allowing agglutinins to be transported to flagellar membrane surfaces, and are also involved in flagellum assembly to regulate gametogenesis [58], [66], [67]. Proteomic analysis of male and female *Plasmodium* gametocytes reveals that kinesin and dynein are proteins expressed in a sex-specific manner [68]. Among the specifically up-regulated genes related to the microtubule-based process in mt+ gametes (Figure 5B), *kinesin motor protein 9-3* (*KIN9-3*, Cre10.g427750), which encodes a kinesin protein, had a significantly lower m^6^A methylation level, indicating that *KIN9-3* is involved in flagellar assembly and associated with sex-specific flagellum formation during mt+ gametogenesis [69]. In addition, qRT-PCR analysis showed a up-regulated trend of *KIN9-3*, *dynein heavy chain 1* (*DHC1*, Cre12.g484250), and *dynein heavy chain 8* (*DHC8*, Cre16.g685450) (Figure 5C).

After the fusion of gametes, a period of light exposure ensures zygote formation and maturation. We detected a global increase in the expression levels of photosynthesis-associated genes in the transition of mt+ and mt− gametes to zygotes, indicating that diploid zygotes may gain energy through photosynthesis (Figure S3B and C). More interestingly, we found that genes with lower expression in zygotes in both mt+ and mt− gametes participate in the microtubule-based process, movement of cell or cellular component, and cilium assembly, indicating that reduced motility in mature zygotes (Figure 5D and E). The identified down-regulated microtubule-related genes with higher m^6^A modification levels are considered candidate genes regulated by m^6^A during the zygotes formation. Among them, *DHC1*, *DHC8, dynein heavy chain 10* (*DHC10*, Cre14.g624950), and *kinesin motor protein 8-1* (*KIN8-1*, Cre13.g602400)*,* as the key genes in the microtubule-based process, were further verified (Figure 5F) [70]. These results suggest that KINs and DHCs are critical for fulfilling sexual reproduction in *Chlamydomonas*.

## Discussion

As the most prevalent mRNA modification in eukaryotes [71], m^6^A modification is involved in many essential biological processes, including cell fate determination [47], [72] and embryo development [47], [73]. In addition, m^6^A modification is important during embryo development and sporogenesis in plants [53], [59]. These findings suggest that m^6^A modulation of mRNA expression may play important roles in the sexual reproduction of both animals and plants. *Chlamydomonas* is an excellent model organism for studying sexual reproduction and life cycle, because it has both characteristics of plants and animals [4]. However, the role and function of m^6^A during this process in *Chlamydomonas* have remained unclear.

During sexual reproduction and the life cycle of *Chlamydomonas*, the cells undergo the transition from a haploid phase to a diploid phase. The vegetative cells and gametes are haploid, while zygotes are diploid. In this study, we performed MeRIP-seq on six samples with duplicates from different stages of *Chlamydomonas* sexual reproduction and life cycle, and compared their transcriptome-wide m^6^A methylomes. Our analyses reveal that m^6^A peaks are markedly enriched in the 3′ UTR, and the distribution pattern of m^6^A is similar among all of the samples. Interestingly, the most enriched sequence motif identified within m^6^A peaks is DRAC, but not the canonical RNA motif of RRACH [63]. The DRAC motif is similar to those characterized in *Arabidopsis* and other eukaryotes. Moreover, by combining MeRIP-seq and RNA-seq, we discovered that the m^6^A level is generally negatively influenced by the abundance of the corresponding mRNAs. Collectively, our data reveal the dynamic changes of m^6^A methylation and the negative correlation between methylation levels with stage-specific transcripts during sexual reproduction and the life cycle of *Chlamydomonas*, suggesting a regulatory role of m^6^A in sexual reproduction.

Previous studies have reported that kinesin and dynein are associated with microtubule-based processes and are important sex-specific expressed proteins, which mediate the intraflagellar transport system and are also involved in flagellar assembly to regulate gametogenesis [58], [67]. In our study, the microtubule-based process and cilium organization from the transition of mt+ vegetative cells to gametes are also specifically accumulated. Among the specifically up-regulated genes in mt+ gametes, *KIN9-3*, *DHC6*, and *KIN16-2* show significantly lower m^6^A methylation levels and are closely related to the microtubule-based process during the mt+ gametogenesis. Additionally, the *DHC1*, *DHC8, DHC10,* and *KIN8-1* genes*,* with lower expression and higher m^6^A methylation levels in zygotes, are also involved in the microtubule-based process. These data together suggest that m^6^A modification potentially participates in sexual reproduction and the life cycle by regulating the abundance of transcripts involved in the microtubule-based movement. Microtubules play important roles in maintaining cell morphology, as well as promoting cell division, signal transduction, and material transport. The flagella structure of *Chlamydomonas* is composed of microtubules. *Chlamydomonas* needs to move rapidly after the generation of gametes to increase the chance of mating. The recognition between different mating types requires the participation of proteins and agglutinins. The m^6^A modification may negatively regulate microtubules and promote the movement of flagella, the transport of substances, and the recognition between gametes.

It is noteworthy that the expression levels of photosynthetic-related genes, including those of PSI, PSII assembly and those involved in light-harvesting and the oxidation–reduction cycle, all decrease during gametogenesis (Figure S3C); this may be related to the nitrogen deficiency treatment to activate gametogenesis program. Similar results have also been observed in a previous study that showed a similar trend in GreenCut2 [24], [74]. Limiting nitrogen leads to decreased photosynthetic activity, carbon assimilation, and chlorophyll biosynthesis in *Chlamydomonas* [75]. The limited substrate and energy could be recycled to synthesize macromolecules required for gametogenesis [75], [76]. In contrast, the down-regulated processes such as photosynthesis and carbohydrate metabolism occurring during the transition of vegetative cells to gametes are up-regulated after the formation of the zygotes from the fusion of gametes (Figure S3B and C). Light plays a key role in the reproductive development of *Chlamydomonas*. Although asexual reproduction may occur in the absence of light, sexual reproduction is entirely dependent on light signals. Photoreceptors regulate the entire process of sexual reproduction in *Chlamydomonas*. For instance, blue light receptors such as Phototropin and Cryptochromes control multiple processes in the sexual reproduction of *Chlamydomonas*. Gametogenesis and zygote germination are regulated by blue and red light, and the occurrence of zygote also requires the presence of blue light receptors [77]. *Arabidopsis* cryptochrome mediates m^6^A modification of more than 10% of mRNAs in the blue light-regulated transcriptome. Cryptochrome interacts with METTL3/METTL14 and promotes the deposition of m^6^A modifications at target genes by liquid–liquid phase separation [78]. Whether m^6^A modification during *Chlamydomonas* sexual reproduction is also regulated by photoreceptors and whether these photoreceptors can also regulate the entire sexual reproduction of *Chlamydomonas* by interacting with m^6^A methyltransferases remain to be further explored.

Taken together, we illustrate the first epitranscriptomic RNA m^6^A profile during the sexual reproduction of *Chlamydomonas*, finding that m^6^A exhibits a conservative distribution pattern and is mainly enriched in the 3′ UTR. More importantly, we found a negative correlation between m^6^A methylation level and gene expression, while m^6^A is likely involved in sexual reproduction through regulating microtubule-based movement. Our study provides new insights into epigenetic regulation in the *Chlamydomonas* life cycle and clues for further studies of m^6^A modification in the evolution of animal and plant reproduction.

## Materials and methods

### Strains and growth conditions

*Chlamydomonas* strains CC-620 and CC-621 were cultivated on a solid TAP medium with photoperiod 12 h light/12 h dark, 22 °C, 50 μmol photons·m^−2^·s^−1^. The cells were resuspended in 60 ml TAP liquid medium with 4–6 × 10^7^ cells per ml for further sample collection.

### Sample preparation

Sample preparation was performed as described previously [24] with slight modifications. Fifteen milliliter *Chlamydomonas* cell suspensions were collected as vegetative cell samples after 2 h under light. The remaining cells were resuspended in 45-ml TAP-N medium, and 15 ml of the cells were harvested as gamete cells after culturing in the light for 21 h, while the remaining 30-ml cells were resuspended in ultrapure water. After being shaken slowly for 30 min under low light, the two strains were mixed in equal amounts and placed under normal light for 2 h to complete the mating. The cell mating status was confirmed by optical microscopic inspection. The zygotes were transferred to a TAP medium containing 3% agar, and half of the cells were collected as a 1d zygote sample after 1 day in the light, while the remaining half was placed in the dark for 6 days and collected as a 7d zygote sample. Before collecting the zygote samples, the lawn on the solid agar surface with vegetative cells was scraped off with a spatula, and the zygotes were collected with a scalpel and resuspended in Tris-EDTA-NaCl (TEN) buffer with 0.2% (v/v) Nonidet P-40 (NP-40) to remove the remaining vegetative cells. After that, the zygotes were collected by centrifugation and resuspended in TEN buffer as previously described.

### Total RNA isolation and mRNA purification

Total RNA was isolated using Trizol (Catalog Nos. 15596026 and 15596018, Invitrogen, Carlsbad, CA) according to the manufacturer’s protocol. The mRNA of *Chlamydomonas* was twice purified using a Dynabeads mRNA Purification Kit (Catalog No. 61006, Ambion, Carlsbad, CA) according to the manufacturer’s instruction to remove the ribosomal RNA as much as possible.

### m^6^A dot-blot assay

An m^6^A dot-blot was performed as described previously [41] with slight modifications. Briefly, the mRNA was serially diluted and loaded as follows: 200 ng, 100 ng, 50 ng, and 25 ng. The mRNA was denatured at 70 °C for 3 min and transferred to a GE Amersham hybond-N^+^ membrane (Catalog No. RPN303B, GE Healthcare, Buckinghamshire, UK) using bio-dot apparatus and a vacuum pump. The mRNA was cross-linked under UV light for 3 min, and the membrane was blocked in PBST with 5% skim milk for 1 h. The membrane with mRNA samples was incubated with a diluted anti-m^6^A antibody in PBST with 5% skim milk overnight. The membrane with mRNA samples was washed with PBST three times for 5 min each time, followed by incubating with the diluted goat anti-rabbit secondary antibody in PBST with 5% skim milk for 1 h. After washing with PBST three times, the membrane with mRNA samples was incubated with enhanced chemiluminescence (ECL) prime Western blotting detection reagent (Catalog No. RPN2232, GE Healthcare) for 1 min and was exposed to film. The membrane with mRNA samples was stained with methylene blue to check the loading amounts.

### MeRIP-seq

m^6^A MeRIP-seq was performed following a previously reported protocol [79]. The 500 ng of purified mRNA was fragmented to a size of about 200 nt using a fragmentation reagent (Catalog No. AM8740, Life Technologies, New York, NY). A total of 30 μl of protein A magnetic beads (Catalog No. 10002D, ThermoFisher Scientific, Waltham, MA) was washed with 1 ml IP buffer [150 mM NaCl, 10 mM Tris-HCl (pH 7.5), 0.1% NP-40 in nuclease-free H_2_O] twice, then resuspended in 500 μl IP buffer with 5 μg anti-m^6^A antibody (Catalog No. ABE572, Millipore, Burlington, MA) mixed in and incubated for at least 6 h at 4 °C with gentle rotation. After two washes with IP buffer [10 mM Tris-HCl (pH 7.4), 150 mM NaCl, and 0.1% NP-40 in DEPC-treated], the mixture of antibodies and beads was resuspended in 500 μl of the IP buffer mixed with 500 ng fragmented mRNA and 5 μl of RNasin plus Rnase inhibitor (Catalog No. N2611, Promega, Madison, WI), which was incubated for 2 h at 4 °C. The beads were then washed twice with 1 ml each of a low-salt IPP buffer [10 mM Tris-HCl (pH 7.5), 50 mM NaCl, 0.1% NP-40 in nuclease-free H_2_O] and a high-salt buffer [10 mM Tris-HCl (pH 7.5), 500 mM NaCl, 0.1% NP-40 in nuclease-free H_2_O] for 10 min at 4 °C. Then, the beads were eluted with 300 μl IPP buffer with 0.5 mg/ml *N*^6^-methyladenosine and RNasin with gentle rotation at room temperature for 1 h. The m^6^A-modified RNAs were eluted with 200 μl of RLT buffer supplied in the RNeasy mini kit (Catalog No. 74106, Qiagen, Hilden, Germany) for 2 min at room temperature. The supernatant was collected in a new tube, and 400 μl of absolute ethanol was added. The mixture was then applied to an RNeasy spin column and centrifuged at 13,000 r/min at 4 °C for 30 s. The spin-column was then washed with 500 μl of RPE buffer supplied in the RNeasy mini kit, and then 500 μl of 80% ethanol, before it was centrifuged at full speed for 5 min at 4 °C to dry the column. The m^6^A-modified RNAs were eluted using 10 μl of nuclease-free H_2_O. For the second round of IP, the eluted RNA was re-incubated using new protein A magnetic beads prepared with a new anti-m^6^A antibody, followed by washing, elution, and purification as described above. The purified RNAs were used for library construction using the KAPA standard RNA-Seq kit (Catalog No. KR1139, KAPA, Boston, MA). The libraries were amplified by PCR for 8–12 cycles and size-selected on an 8% TBE gel. Sequencing was carried out by the Illumina Nova 6000 platform.

### Sequencing data analysis

Pair-end reads with a length of 150 bp were generated by MeRIP-seq and RNA-seq. Cutadapt (version 1.16) [80] software and Trimmomatic (version 0.33) [81] were used to trim adapters and low-quality sequences for raw reads. The remaining reads were aligned to the *Chlamydomonas* genome (version 5.6 for assembly; Phytozome version 12 for gene annotation) using Hisat2 (version 2.0.5) [82]. Only uniquely mapped reads with mapping quality scores ≥ 20 were used for the subsequent analysis. The number of reads mapped to genes (Phytozome version 12) was counted using the software featureCounts (version 1.6.2) [83]. The genes with reads per kilobase per million mapped reads (RPKM) > 1 in both replicates as the expressed genes. For MeRIP-seq, the replicates were merged for calling m^6^A peak using R package exomePeak [84], with the corresponding input samples serving as control. The software BEDTools’ intersectBed (version 2.28.0) [85] was used to annotate each m^6^A peak based on the gene annotation information.

### Statistical analysis of DEGs and GO analysis

DEGs among different samples were determined using the R package edgeR [86]. Transcripts with |log_2_ fold change| > 1 and FDR < 0.01 were considered DEGs. GO analysis of a specific gene set was performed using agriGO [87] (https://systemsbiology.cau.edu.cn/agriGOv2/). GO terms with *P* < 0.05 were considered statistically significant.

### Identification of differential m^6^A peaks and motifs within m^6^A peaks

To identify the differential m^6^A peaks, we calculated the fold change of enrichment between different stages and calculated the differential significance using the reads mapped to IP and input from each sample [88]. The peaks with |log_2_ fold change| > log_2_ 1.5 and *P* < 0.05 (chi-square test) were considered as differential peaks. HOMER (version 4.7) [89] was used to identify the motif enriched by m^6^A peaks, and the motif length was limited to 5 nt. The peaks annotated to mRNA were considered target sequences, and the background sequences were constructed by randomly perturbing these peaks using shuffleBed from BEDTools (version 2.28.0) [85].

### qRT-PCR analysis

RevertAid first strand cDNA synthesis kit (Catalog No. K1622, ThermoFisher Scientific) was applied to generate cDNA templates by reverse transcription. The TB green premix Ex taq (Catalog No. RR420A, TaKaRa, Kyoto, Japan) was used in the qRT-PCR reaction, and the qRT-PCR was carried out using LightCycler480 (Roche). The *guanine nucleotide-binding protein beta subunit-like* (*CBLP*, Cre06.g278222) gene was used as the internal control. The calculation of relative mRNA expression was performed as described previously [90].

## Data availability

The raw sequence data from RNA-seq and MeRIP-seq have been deposited in the Genome Sequence Archive [91] at the National Genomics Data Center, Beijing Institute of Genomics, Chinese Academy of Sciences / China National Center for Bioinformation (GSA: CRA005106), and are publicly accessible at https://ngdc.cncb.ac.cn/gsa.

## Competing interests

The authors have declared no competing interests.

## CRediT authorship contribution statement

**Ying Lv:** Conceptualization, Methodology, Investigation, Writing – original draft. **Fei Han:** Resources, Investigation, Writing – original draft. **Mengxia Liu:** Software, Data curation, Writing – original draft. **Ting Zhang:** Methodology, Data curation. **Guanshen Cui:** Resources, Investigation. **Jiaojiao Wang:** Software, Validation. **Ying Yang:** Methodology, Writing – review & editing. **Yun-Gui Yang:** Conceptualization, Writing – review & editing. **Wenqiang Yang:** Conceptualization, Writing – review & editing, Supervision. All authors have read and approved the final manuscript.

## Supplementary material

Supplementary Table S1 **The identified m6A peaks at different stages of life cycles**.

Supplementary Table S2 **Gene expression reads per kilobase per million mapped reads (RPKM) matrix**.

Supplementary Table S3 **The differentially expressed genes at different stages**.
